# Relationship Between Membrane Vesicles, Extracellular ATP and Biofilm Formation in Antarctic Gram-Negative Bacteria

**DOI:** 10.1007/s00248-020-01614-6

**Published:** 2020-10-06

**Authors:** Nicolas Baeza, Elena Mercade

**Affiliations:** Secció de Microbiologia, Departament de Biologia, Sanitat i Medi Ambient, Universitat de Barcelona, Barcelona, Spain

**Keywords:** Membrane vesicles, Gram-negative bacteria, Biofilm, Extracellular ATP, Antarctica

## Abstract

Biofilms offer a safe environment that favors bacterial survival; for this reason, most pathogenic and environmental bacteria live integrated in biofilm communities. The development of biofilms is complex and involves many factors, which need to be studied in order to understand bacterial behavior and control biofilm formation when necessary. We used a collection of cold-adapted Antarctic Gram-negative bacteria to study whether their ability to form biofilms is associated with a capacity to produce membrane vesicles and secrete extracellular ATP. In most of the studied strains, no correlation was found between biofilm formation and these two factors. Only *Shewanella vesiculosa* M7^T^ secreted high levels of extracellular ATP, and its membrane vesicles caused a significant increase in the speed and amount of biofilm formation. In this strain, an important portion of the exogenous ATP was contained in membrane vesicles, where it was protected from apyrase treatment. These results confirm that ATP influences biofilm formation. Although the role of extracellular ATP in prokaryotes is still not well understood, the metabolic cost of its production suggests it has an important function, such as a role in biofilm formation. Thus, the liberation of extracellular ATP through membrane vesicles and its function deserve further study.

## Introduction

Gram-negative bacterial membrane vesicles (MVs) have been the subject of numerous studies in recent years [1, 2]. While there is growing evidence that more than one type of MV exists [3], MVs produced by Gram-negative bacteria are described as small (20–300 nm), spherical, bilayered membranous structures, derived from the outer membrane of the cell [4]. MV components, which can vary according to the strain and mechanism of formation, are mainly outer membrane lipids and proteins and periplasmic elements; inner membrane proteins and cytoplasmic compounds such as proteins, DNA or RNA may also be present [1, 5]. Along with this diversity of components, MVs are involved in several functions related to bacterial survival, pathogenicity, interaction with the host immune system, intercellular communication, and genetic transfer [1, 6, 7].

Both planktonic and sessile bacterial cells in biofilms can produce MVs [8]. Several studies have shown that MVs are ubiquitous in the extracellular matter of many Gram-negative bacterial biofilms [9, 10], and our group demonstrated the presence of huge amounts of MVs interspersed in the exopolymeric substance (EPS) secreted by cold-adapted Antarctic strains [11, 12].

Wang and co-workers reviewed the available data on the link between MVs and biofilms [13], which has been mainly analyzed in pathogenic bacteria. Several studies indicate that different MV components are essential for biofilm formation and maintenance, but their individual roles are difficult to elucidate due to the compositional complexity of MVs. For a specific strain of *Helicobacter pylori*, it was suggested that a 22-kDa protein was involved in biofilm formation [14]. MVs from *Vibrio cholera* El Tor C6706 contained the DegP protein, which enhanced biofilm formation [15]. Alterations in O-polysaccharide from the LPS of *Pseudomonas aeruginosa* PAO can limit biofilm maturation [16], and the mere presence of MVs in *Francisella* biofilm suggests their involvement in its formation [17].

Although pathogenic bacterial biofilms are a serious concern in terms of health, biofilms from environmental strains also play a key role in nature. The presence of MVs in biofilms from environmental bacteria has been described [8, 18], but the role of MV components in their formation is less studied.

As adenosine triphosphate (ATP) has been found in MVs [19], it could be related to biofilm formation [20], and is therefore an interesting component for analysis. It is well known that ATP plays a crucial role inside all living cells as an energy source for many essential reactions [21]. Moreover, in eukaryotic cells, ATP can cross the plasma membrane by different mechanisms and influence several physiological processes through the purinergic signaling system [22]. For example, eukaryotic cells damaged by bacterial infection can release extracellular ATP (eATP) [23], which acts as a danger molecule, interacting with P2 receptors to elicit an immune response against the pathogen. Bacterial cells can respond to this attack by increasing or modifying biofilm formation to protect themselves from liberated immune cells and cytokines [20, 24, 25]. Bacteria can also respond to an immune system attack or damage by other stress factors such as virus infection or antibiotic presence by increasing the production of MVs [26–29]. Several studies have analyzed the release of eATP in prokaryotic cells, but its function and the mechanisms of secretion are still underexplored [30, 31].

Ivanova and co-workers reported that many heterotrophic bacterial species released eATP during attachment to different surfaces [32]. Similarly, Mempin and co-workers demonstrated that many bacteria released eATP to the external medium in a growth phase-dependent manner to perform a physiological function [33].

Our research group has worked for a long time with a set of cold-adapted Antarctic strains. In previous studies, we determined that these strains produce large amounts of exopolymers as a cold adaptation strategy, which could enable them to form biofilms [34]. Additionally, many of these Antarctic strains presented complex extracellular matter containing huge amounts of MVs, which in some cases included ATP [11, 12, 19, 34].

Based on these findings, in this work, we used a set of Antarctic cold-adapted Gram-negative strains to analyze whether there is a relationship between their ability to produce MVs and secrete eATP, and also to assess the potential role of both factors in biofilm development. The only strain showing such a relationship was *S. vesiculosa* M7^T^, which was used to study the effect of MV supplementation on biofilm formation. The secretion of MV-associated ATP and its influence on the formation of biofilms were also analyzed.

## Material and Methods

### Bacterial Strains

The strains used in this study are described in Table 1. All strains were isolated and characterized from Antarctic samples in our facilities and are cold-adapted strains. The strains were routinely maintained on Trypticase Soy Agar (TSA, Oxoid), and stored at − 80 °C in Cryoinstant tubes (Deltalab, S.L). For MV isolation and supernatant ATP determination, all strains were grown in Trypticase Soy Broth (TSB, Oxoid). Unless otherwise stated, cultures were incubated for 48 h at 15 °C in an orbital shaker (innova® 44, New Brunswick Scientific) at 180 rpm. When necessary, growth was monitored by measuring the optical density (OD) at 600 nm or by counting colony-forming units (CFU/ml) using the plating serial dilution method on TSA plates (CFU/ml).

**Table 1:** Bacterial strains used in this study

Species/strain	Collection no.	Isolation site	Reference
*Shewanella vesiculosa* M7^T^	LMG 24424	Marine sediment, Deception Island, Antarctica	Bozal et al. (2009) IJSEM. 59:336–340
*Shewanella frigidimarina* NF12	LMG 19867	Glacier mud, King George Island, Antarctica	Bozal et al. (2002) IJSEM. 52:195–202
*Shewanella livingstonensis* NF22^T^	LMG 19866	Water, Livingston Island, Antarctica	Bozal et al. (2002) IJSEM. 52:195–202
*Psychrobacter fozii* NF23^T^	LMG 21280	Marine sediment, King George Island, Antarctica	Bozal et al. (2003) IJSEM. 53:1093–1100
*Psychrobacter glacincola* NF1	LMG 21282	Glacier mud, King George Island, Antarctica	Own collection
*Psychrobacter immobilis* NF18	LMG 21277	Water, Livingston Island, Antarctica	Own collection
*Psychrobacter luti* NF11^T^	LMG 21276	Glacier mud, King George Island, Antarctica	Bozal et al. (2003) IJSEM. 53:1093–1100
*Pseudomonas sp.* ID1	–	Sediment, Deception Island, Antarctica	Own collection
*Pseudoalteromonas* M2	–	Sediment, Deception Island, Antarctica	Own collection
*Pseudoalteromonas* M4–1	–	Sediment, Deception Island, Antarctica	Own collection
*Pseudomonas aeruginosa* PAO1	–	–	–

### MV Isolation

MVs were collected from cultures using a previously reported method [35]. In brief, each strain was cultured in 2-L marked flasks with 500 ml of TSB. Unless otherwise stated, cultures were incubated for 48 h at 15 °C in an orbital shaker (innova® 44, New Brunswick Scientific) at 180 rpm. Flasks were inoculated with 1% of pre-inoculum from a 50-ml culture of TSB grown overnight at 15 °C. MVs were isolated when cultures of each strain reached an OD_600_ = 2.2. For that purpose, cells were pelleted by centrifugation at 10,000 rpm for 30 min at 4 °C, and the supernatant was filtered through 0.45-μm pore-size filters to remove remaining bacterial cells. MVs were obtained by centrifugation at 44,000×*g* for 1 h at 4 °C in an Avanti® J-20 XP centrifuge (Beckman Coulter, Inc). Pelleted MVs were resuspended in 50 ml of Dulbecco’s phosphate-buffered saline (PBS, Gibco, Life Technologies) and filtered through 0.22-μm pore-size Ultrafree spin filters (Millipore). After that, MVs were pelleted again and resuspended in a minimal volume of PBS.

### MV Quantification

MVs were quantified by measuring their lipopolysaccharide (LPS) content using the Purpald assay [36]. The standard test curve ranged from 0.05 to 0.4 mM of 2-keto-3-deoxyoctonate (Sigma-Aldrich). To 50 μl of each sample or standard, 50 μl of 32 mM NaIO_4_ (Sigma-Aldrich) was added, and the culture was incubated for 25 min, followed by the addition of 50 μl of 136 mM of Purpald® (Sigma-Aldrich) reagent in 2 N NaOH. After a further 20 min of incubation, 50 μl of 64 mM NaIO_4_ was added and the culture incubated for 20 min. Foam was eliminated from each well by the addition of 20 μl of 2-propanol. Absorbance (550 nm) was measured in a Modulus Microplate Multimode Reader (Turner Biosystems). LPS concentration of samples was normalized to 1 L of bacterial culture.

### High-Pressure Freezing and Freeze-Substitution (HPF-FS) of Strains and TEM Observation

For transmission electronic microscopy (TEM), bacteria were grown on TSA for 3 days at 15 °C. TEM observations of the strains and their MVs were performed as described by Pérez-Cruz et al. [37]. In brief, bacterial colonies were transferred to planchettes and immediately cryoimmobilized using a Leica EMPact high-pressure freezer (Leica, Vienna, Austria). Frozen samples were freeze-substituted in a Leica EM AFS system (Leica, Vienna, Austria), where the substitution was performed in pure acetone containing 2% (w/v) osmium tetroxide and 0.1% (w/v) uranyl acetate at − 90 °C for 72 h. The temperature was increased (Δ*t* = 5 °C/h) to 4 °C and held constant for 2 h, and finally increased to room temperature and maintained for 1 h. Samples were washed in acetone at room temperature and infiltrated in a graded series of Epon-acetone mixtures (Epon 812, Ted Pella, Inc., USA) for 30 h. Samples were embedded in fresh Epon and polymerized at 60 °C for 48 h. Ultrathin sections were cut with a Leica UCT ultramicrotome and mounted on Formvar carbon-coated copper grids. Sections were post-stained with 2% (w/v) aqueous uranyl acetate and lead citrate. Samples were examined in a Tecnai Spirit electron microscope (FEI Company, Netherlands) at an acceleration voltage of 120 kV.

### ATP Quantification

In order to determine eATP secreted into the medium, each strain was cultured in 50 ml of TSB at 15 °C and 180 rpm until OD_600_ = 2.2. Then, 1 ml of each culture was centrifuged at 10,000 rpm for 30 min at room temperature and the supernatant was filtered by 0.45 μm pore-size Ultrafree spin filters (Millipore) and transferred to a fresh tube. eATP levels of the samples were measured using the BacTiter-Glo™ Microbial Cell Viability Assay Reagent (Promega, Madison, WI), based on the luciferase reaction. ATP levels were determined by measuring luminescence levels. Sample values were extrapolated to an adenosine 5′-triphosphate sodium salt (Promega) standard curve of tenfold dilutions of ATP from 1 μM to 10 pM. For each point of the curve, 100 μl of the supernatant was mixed with 100 μl of BacTiter-Glo™ Cell Viability Assay Reagent in a Corning® 96-well white polystyrene plate (Merck), and incubated at room temperature for 30 min. Luminescence was measured in a Modulus Microplate Multimode Reader (Turner Biosystems). Sterile-filtered TSB (Oxoid) was used as a blank. All quantifications were carried out in triplicate in three independent experiments.

For the *S. vesiculosa* M7^T^ strain, intracellular ATP in cells (icATP) and eATP in the supernatant, were measured along the growth curve. ATP levels were determined every 2 h during the logarithmic phase and every 4 h during the stationary phase. From a 500-ml culture, 600 μl-aliquots were collected at the aforementioned times to prepare bacterial extracts using the perchloric acid extraction method [38]. A 600 μl-aliquot was mixed with 300 μl of ice-cold 1.2 M perchloric acid and vortexed for 10 s. The mixture was incubated on ice for 15 min and centrifuged at 10,000 rpm for 5 min at room temperature. Six hundred microliters of the supernatant was transferred to a fresh tube and mixed with 300 μl of a neutralizing solution containing 0.72 M KOH and 0.16 M KHCO_3_. The neutralized extract was centrifuged at 10,000 rpm for 5 min at room temperature and the supernatant was transferred to a fresh tube for the assay. From the same culture and at the same times, samples of 1 ml were collected and centrifuged at 10,000 rpm for 30 min. Supernatants were filtered by 0.45-μm pore-size Ultrafree spin filters (Millipore) and transferred to fresh tubes. As described above, 100 μl of the supernatants was assayed to determine the ATP level. Sterile-filtered TSB was used as a blank. All quantifications were done in triplicate in three independent experiments.

To determine if ATP was inside the MVs, the vesicles were isolated at an OD = 2.2 as described above and split into three equal aliquots. The first aliquot was diluted 1:100 in PBS, centrifuged (44,000*g*, 1 h, 4 °C), and then treated with 0.1% Triton X-100 to lyse the MVs and make ATP available for measuring. The second aliquot was treated with the enzyme apyrase (2 μg/ml, 30 min, 30 °C) to deplete potential ATP present outside the MVs. The apyrase was eliminated by diluting 1:100 in PBS and centrifuging (44,000*g*, 1 h, 4 °C). Then MVs were lysed with 0.1% Triton X-100. Finally, in the third aliquot, MVs were first lysed with 0.1% Triton X-100 and then treated with apyrase (2 μg/ml, 30 min, 30 °C) to eliminate ATP. The ATP concentration of all three aliquots was measured by the luminescence assay as described above. All quantifications were done in triplicate in three independent experiments.

### Biofilm Quantification

To quantify biofilm formation, the strains listed in Table 1 were first cultivated in 50-ml flasks of TSB until OD_600_ = 2.2. Cultures were diluted 1:100 in sterile TSB and added to TPP® 96-well culture plates (Merck) at 200 μl per well in eight replicates. Sterile TSB was used as a negative control. *P. aeruginosa* PAO1 culture was used as a positive control for biofilm formation. Plates were incubated for 6 days at 15 °C for the Antarctic strains and at 37 °C for *P. aeruginosa* PAO1, without shaking. After incubation, biofilm formation was measured by the crystal violet method [39]. In brief, cultures were removed from the wells and washed three times with 200 μl of sterile water. Then, 200 μl of a 0.1% solution of crystal violet in water was added to each well and left for 30 min at room temperature. Plates were again washed with water three times and left to dry for 1 h at 37 °C. Once dried, 200 μl of acetic acid (30% v/v) was added to each well and left for 30 min at room temperature with mild shaking. Finally, absorbance was quantified in a Modulus Microplate Multimode Reader at 570 nm.

The results were interpreted using the method proposed by Stepanovic et al. [40], in which a threshold is established at which the biofilm is formed. A strain is biofilm-forming if the OD levels obtained exceed the OD resulting from the negative control plus three times the standard deviation (SD) of said negative control [OD threshold = OD negative control + 3 × SD]. The strains are divided into 4 categories, depending on the levels of biofilm formation: (1) the strain is not biofilm-forming (−) if the OD obtained is less than or equal to the OD threshold; (2) biofilm formation is weak (+) if the OD obtained is greater than and less than twice the threshold OD; (3) moderate biofilm formation (++) is indicated by an OD greater than two times and less than four times the threshold OD; and (4) a strong producer of biofilms (+++) is indicated by an OD greater than four times the threshold OD.

### Effect of MVs on Biofilm Formation

To determine the effect of MVs on biofilm formation in *S. vesiculosa* M7^T^, the inoculum and biofilm development were performed as described above. For the cultures supplemented with MVs, isolated MVs were diluted to 0.57 mg/ml and 0.057 mg/ml of LPS, and then 20 μl was added to the 200 μl of inoculated TSB to a final concentration of 0.057 and 0.0057 mg/ml of LPS, respectively. *S. vesiculosa* M7^T^ cultures without added MVs were used as a standard biofilm control, and wells with added sterile TSB were used as a negative control. Cultures were incubated for 1, 2, 3, 4, 5, and 6 days at 15 °C without shaking, and biofilm formation was quantified by the crystal violet method. Four independent experiments were carried out with eight replicas of each assayed condition.

To determine cell aggregation levels in biofilms and MV-supplemented biofilms, cultures were performed as previously described but using 24-well plates with a glass coverslip at the bottom of each well. After 1, 2, 3, 4, 5, and 6 days of incubation at 15 °C without shaking, biofilms were stained with 0.1% crystal violet. Once dried, the coverslips were attached to the slides and observed by bright field microscopy using a Leica DM 1000 microscope. Images were taken with a Leica DFC290 microscope camera. For each day, biofilm images (*n* = 20) were analyzed with ImageJ Fiji software [41]. A scale for all images was set, a threshold was adjusted to differentiate between cells and background, and holes between cells were filled for homogeneity. Three different sizes of aggregation were established to define biofilm development: single cells, small cell clusters made up of dozens of cells, and large cell clusters made up of hundreds and thousands of cells often arranged in more than one layer.

To determine the effect of MV integrity on biofilm formation, the vesicles were broken after three freeze-thaw cycles. Broken MVs were added at the final concentration of 0.057 mg/ml. Additionally, to study the effect of the apyrase treatment, the enzyme was added (2 μg/ml for 30 min at 30 °C) to wells containing intact or broken MVs (0.057 mg/ml). Two independent experiments were carried out with eight replicas of each assayed condition.

### Effect of ATP on Biofilm Formation

To determine the effect of ATP on biofilm formation in *S. vesiculosa* M7^T^, inoculum and biofilm development was carried out as described above. Cultures were supplemented with free ATP (Promega) to final concentrations of 1 nM and 0.1 nM and a final volume of 200 μl. A non-ATP-supplemented *S. vesiculosa* M7^T^ culture was used as a positive control and sterile TSB (Oxoid) as a negative control. Cultures were incubated for 6 days at 15 °C without shaking, and biofilm formation was quantified by the crystal method as described above. Three independent experiments were carried out with eight replicas of each assayed condition.

### Statistics

Statistical analysis of the data was performed using StatGraphics XVII Version 17.2.07 (64-bit) (Statgraphics Technologies, Inc., The Plains, Virginia). A two-tailed *T* test was performed to analyze the mean differences between supplemented biofilm treatments and the control, and the differences in the ATP levels of the three differently treated aliquots. The level of significance was set at *P* = 0.05 for all tests.

## Results

### Relationship between MVs, eATP, and Biofilm Formation

**Fig. 1:**
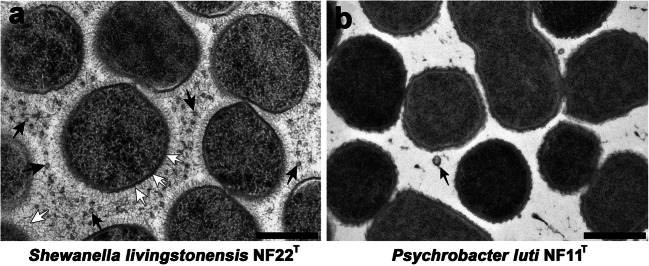
Representative TEM images of Antarctic strain sections after high-pressure freezing and freeze substitution (HPF-FS). **a** *S. livingstonensis* NF22^T^. **b** *P. luti* NF11. Black arrows indicate MVs present in the extracellular matter. White arrows indicate fibers from the capsular material and dispersed EPS. Bars are 500 nm

A collection of cold-adapted Antarctic strains isolated and characterized in our laboratory was used to study the relationship between MVs, eATP, and biofilm formation in Gram-negative bacteria (Table 1). To ascertain the ability of these strains to produce MVs, vesicles were retrieved from the culture media (OD = 2.2) by filtration of cell-free supernatants and high-speed centrifugation. The MV concentration was determined by measuring LPS, a compound exclusive to the outer membrane layer of Gram-negative bacteria, using the Purpald assay protocol. A wide variety of MV concentrations was observed among the analyzed strains, ranging from 3.421 mg/ml for *Shewanella livingstonensis* NF22^T^ to 0.0011 mg/ml for *Psychrobacter immobilis* NF18 (Table 2). To confirm the presence of MVs, each strain was analyzed by TEM after high-pressure freezing and freeze substitution. TEM observation also revealed a highly variable presence of MVs in the extracellular matter from the different strains, but a good correlation was found between the LPS concentration and the amount of MVs visualized. Representative images from two strains can be observed in Fig. 1. In *S. livingstonensis* NF22^T^, which had the highest value of LPS (3.421 mg/ml), a very large amount of MVs was seen spread among the cells (Fig. 1a, black arrows), whereas in *Psychrobacter luti* NF11^T^, which had a very low LPS content (0.073 mg/ml), MVs were observed only sporadically in the extracellular matter (Fig. 1b, black arrow). Interestingly, bacteria from the same genus showed variable LPS concentrations, implying that they produced different amounts of MVs. Additionally, for some strains such as *S. livingstonensis* NF22^T^, long fibers that can be described as capsular material were visualized around the cells perpendicular to the outer membrane (Fig. 1a, white arrows). Many strains also showed a netlike mesh of fibers in the extracellular matter corresponding to exopolymeric substances secreted by cells (Fig. 1a, white arrow).

**Table 2:** Capacity of Antarctic strains to produce MVs, form biofilms and secrete eATP. To determine MV concentration and eATP, strains were grown on TSB for 48 h at 15 °C and 180 rpm. For biofilm formation, strains were grown on 96-well plates at 15 °C without shaking for 6 days

Strain	MV^a^ (mg/ml LPS)	Biofilm^b^	eATP^c^
*Shewanella vesiculosa* M7^T^	2.573	++	++
*Shewanella frigidimarina* NF12	1.098	+	–
*Shewanella livingstonensis* NF22^T^	3.421	+	–
*Psychrobacter fozii* NF23^T^	0.014	+	+++
*Psychrobacter glacincola* NF1	0.001	+	+
*Psychrobacter immobilis* NF18	0.001	+++	++
*Psychrobacter luti* NF11^T^	0.073	+++	+
*Pseudomonas sp.* ID1	0.065	+++	–
*Pseudoalteromonas* M2	–	+	++
*Pseudoalteromonas* M4–1	2.051	+	–
*Pseudomonas aeruginosa* PAO1	ND	++	ND

^a^MVs were collected from 1 L cultures and quantified by LPS concentration using the Purpald Assay protocol

^b^Biofilm formation was determined by the crystal violet method. To interpret the results, an OD threshold at which biofilm is formed was established. A strain is biofilm-forming if OD levels exceed the OD of the negative control plus three times its standard deviation (SD) [OD threshold = OD negative control + 3 × SD]. Strains can be divided into 4 categories: the strain is not biofilm-forming (−) if the OD is less than or equal to the OD threshold; a weak biofilm producer (+) if the OD is greater than and less than twice the OD threshold; moderately biofilm-forming (++) if the OD is greater than twice and less than four times the OD threshold; and a strong biofilm producer (+++) if the OD is greater than four times the OD threshold

^c^eATP from cultures (OD = 2.2) was determined from 0.22 μm-filtered supernatant broths and bioluminescence was measured by a BacTiter-Glo Microbial Cell Viability Assay (Promega). For a simpler interpretation, “−”was assigned to bacteria that exported less than 1 nM to the supernatant; “+” between 1 nM and 10 nM; “++” between 10 nM and 100 nM; and “+++” for more than 100 nM

The ability to form biofilms by the same group of strains was evaluated by growing them for 6 days in 96-well culture plates and measuring biofilms by the crystal violet method. For most strains, no correlation was detected between the ability to form biofilms and the capacity to secrete MVs to the medium, except for the strain *S. vesiculosa* M7^T^ (Table 2). For example, *Psychrobacter immobilis* NF18, which produced very low amounts of MVs (0.0011 mg/ml LPS), exhibited a high capacity to form biofilms (+++). Conversely, *S. livingstonensis* NF22^T^, which produced the greatest number of MVs (3.421 mg/ml LPS), developed only a small amount of biofilm (+). *S. vesiculosa* M7^T^ was the only strain that secreted high amounts of MVs (2.573 mg/ml LPS) and at the same time developed a detectable biofilm (++) similar to that obtained with the control strain *P. aeruginosa* PAO1.

It is worth noting that colonies from all strains had a mucoid appearance, especially *P. deceptionensis* ID1, whose biofilm formation values were 3.5 times higher than the other strains.

The third parameter was the presence of eATP in filtered supernatants of the cultured strains analyzed by an assay based on the luciferase reaction. For all the assayed strains with the exception of *S. vesiculosa* M7^T^, no correlation was found between the presence of eATP, biofilm formation, and MV secretion (Table 2). *P. fozii* NF23^T^ secreted the highest amount of eATP to the medium, but it produced low levels of MVs (0.014 mg/ml) and formed weak biofilms (+). Liberation of eATP to the medium did not depend on the bacterial genus analyzed; the three *Psychrobacter* strains produced different amounts. The same was observed for the strains of the genera *Shewanella*, *Pseudomonas*, and *Pseudoalteromonas*. *S. vesiculosa* M7^T^ gave intermediate values for eATP (++) and biofilm formation (++), while producing high amounts of MVs, and was therefore chosen for further study of the potential relationship between the three factors.

### Effect of MV Addition on *S. vesiculosa* M7^T^ Biofilm Formation

**Fig. 2:**
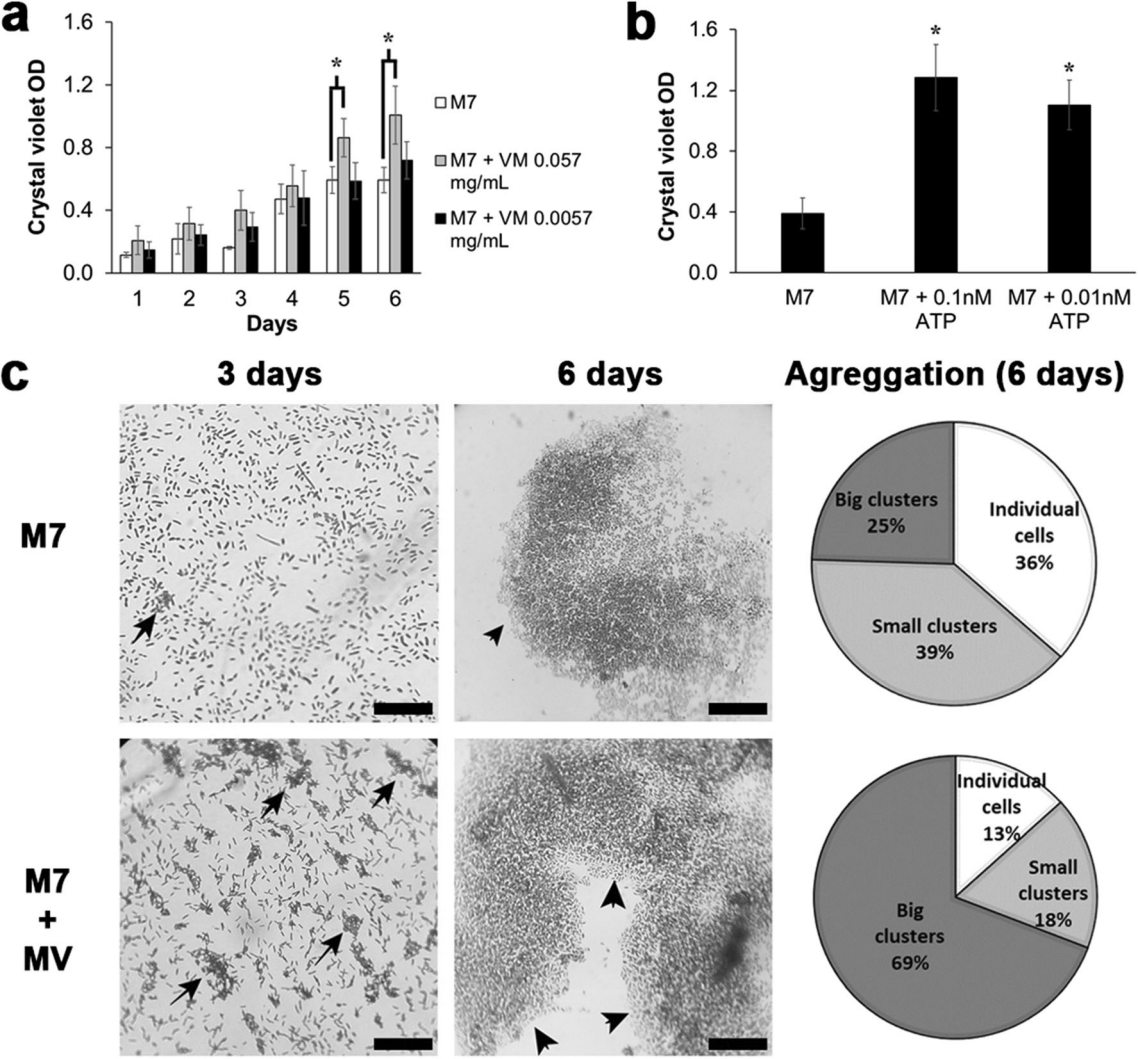
Biofilm formation by *S. vesiculosa* M7^T^ supplemented with MVs or ATP. **a** The effect of the addition of MVs. *n* = 4, ^*^P < 0.05 M7 control vs M7 MV-supplemented cultures (0.057 mg/ml). **b** The effect of exogenous ATP addition on biofilm formation. *n* = 3, ^*^P < 0.05 vs M7 control. In both **a** and **b**, biofilm formation was determined in TSB in 96-well microtiter plates at 15 °C without shaking using the crystal violet method (OD 570 nm). **c** Images of optical microscopy showing distribution of adhered *S. vesiculosa* M7^T^ cells with and without MV supplementation at different incubation times. Circles show percentages of the different types of aggregation obtained using ImageJ at 6 incubation days. Bars are 20 μm

To determine the effect of MV addition on *S. vesiculosa* M7^T^ biofilm formation, cultures were supplemented with MVs previously isolated from the strain, and diluted to the final concentrations of 0.057 and 0.0057 mg/ml of LPS. A non-MV-supplemented culture of *S. vesiculosa* M7^T^ was used as a control for biofilm formation and sterile TSB (Oxoid) as a negative control. The cultures were incubated for 1, 2, 3, 4, 5, and 6 days, and biofilm formation was quantified by the crystal violet method. As can be observed in Fig. 2a, from the first day it was detectable that the addition of 0.057 mg/ml of MVs favored biofilm formation, and these differences became significant at days 5 and 6. However, the lowest concentration of MVs had no significant influence on biofilm formation at any day.

To visualize biofilm formation, with and without MV addition, the same experiment was repeated but this time *S. vesiculosa* M7^T^ cells were grown on coverslips placed at the bottom of 24-well plates to allow cell adherence on the glass. After incubation, the coverslips were removed and stained with crystal violet and the cells were observed under the microscope. Adhesion of *S. vesiculosa* M7^T^ cells on glass coverslips was visualized after 1 day of incubation. Microscopy analysis showed single cells attached to the glass surface in both samples, with and without added MVs (0.057 mg/ml). Cells were uniformly distributed, and the number of attached cells increased gradually over 1 to 3 days of incubation. After 3 days, a higher number of microcolonies formed by dozens of clustered cells were observed in samples with added MVs at 0.057 mg/ml (Fig. 2c, see arrows). These small cell groups, ranging from 5 to 15 μm in size, could be considered as points of incipient biofilm formation. In contrast, in the samples of *S. vesiculosa* M7^T^ grown without MV supplementation, isolated adhered cells were distributed regularly and very few microcolonies were seen. After 5–6 days, cell distribution in samples with and without MVs had undergone significant changes. In both samples, *S. vesiculosa* M7^T^ formed large cell clusters considered as biofilms (Fig. 2c, arrowheads). To quantify differences in cell adhesion and aggregation between the samples at day 6, Image J software was used and three groups were defined: samples with individual cells, small cell clusters (microcolonies) or large cell clusters (biofilms). Counting the types of aggregation on each glass coverslip confirmed a significant increase in biofilm formation when *S. vesiculosa* M7^T^ was incubated in the presence of MVs (Fig. 2c): the majority of cells were grouped in large clusters, which had a patchy distribution across the glass (69%); 13% of cells were attached individually and 18% formed small clusters. However, in *S. vesiculosa* M7^T^ incubated without MVs, a large part of the cells was still adhered individually (36%), another portion formed small clusters (39%) and only 25% were observed as biofilm groups.

In order to verify the effect of ATP on *S. vesiculosa* M7^T^ biofilm formation, 96-well microtiter plate TSB-cultures were supplemented with two ATP concentrations (0.1 nM, 0.01 nM) and incubated for 6 days at 15 °C without shaking. Biofilm formation was then measured and compared with that of the *S. vesiculosa* M7^T^ strain. The concentrations of ATP were the same as those of eATP present in the two concentrations of MVs previously used. All the added eATP concentrations caused a significant increase in biofilm formation (Fig. 2b).

### Presence of eATP in *S. vesiculosa* M7^T^ Cultures

**Fig. 3:**
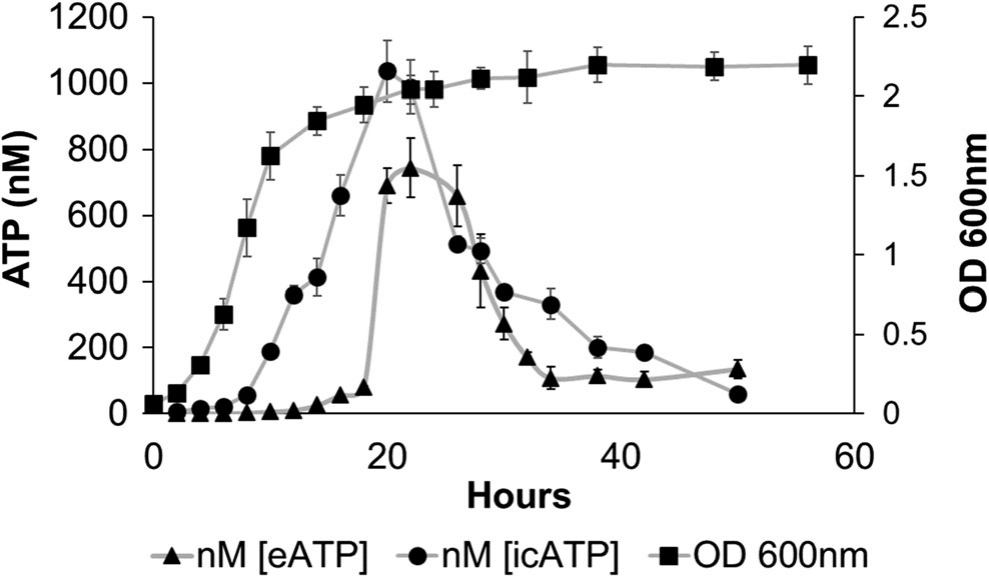
Growth curve of *S. vesiculosa* M7^T^ and levels of intracellular icATP and extracellular eATP during growth. *S. vesiculosa* M7^T^ was cultured in TSB at 15 °C and 180 rpm. Aliquots were taken and OD_600_ was measured at every point. Aliquots were centrifuged at 10,000 rpm and filtered (0.22 μm). ATP concentration from supernatants was measured with the BacTiter-Glo Microbial Cell Viability Assay (Promega). *n* = 2

To measure the eATP secreted by *S. vesiculosa* M7^T^ during growth, TSB cultures were grown. OD was used to follow the growth, and the ATP concentration was measured inside cells (icATP) and in filtered supernatants at the same growth points (eATP) (Fig. 3). After 24 h, when cells reached the end of the exponential phase and entered the stationary phase (OD = 2), the highest concentration of icATP (1036.0 nM) was observed. Similarly, the eATP concentration reached a maximum value at 24 h of cultivation and then progressively decreased, similarly to the icATP levels (Fig. 3).

### eATP Is Contained Inside MVs from *S. vesiculosa* M7^T^

**Fig. 4:**
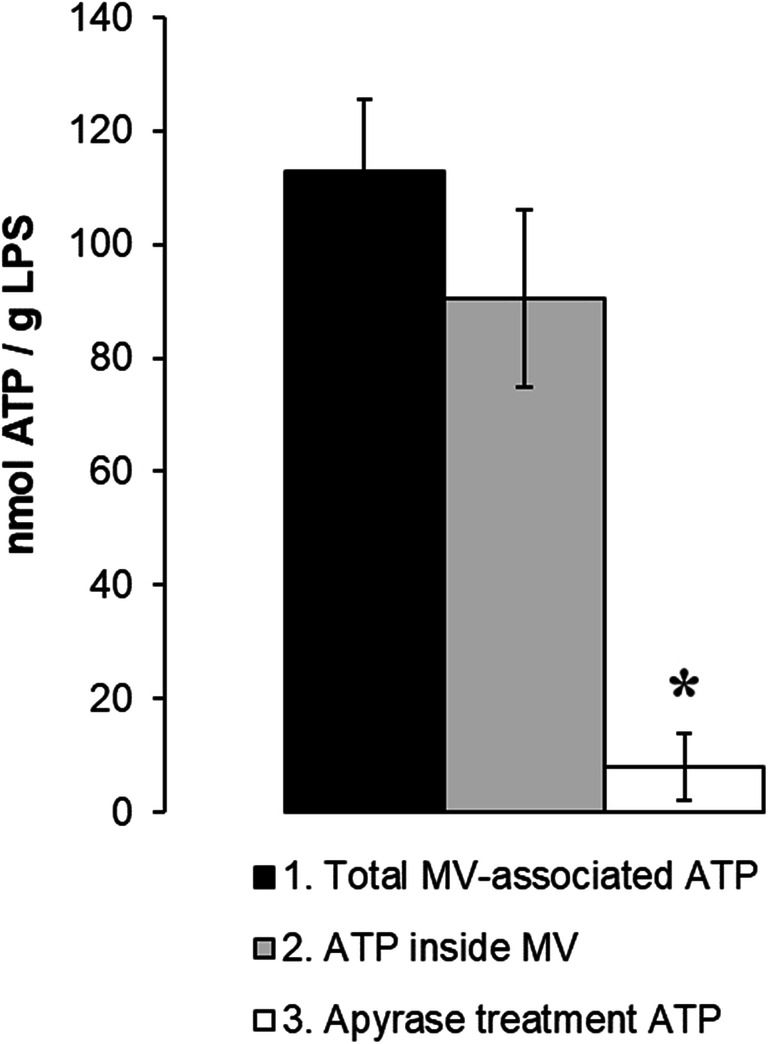
Extracellular ATP is packaged inside MVs from of *S. vesiculosa* M7^T^. MVs were collected from a 48-h TSB culture at 15 °C and 180 rpm. MVs were split into three equal aliquots: one was treated with 0.1% Triton X-100 to lyse the MVs and measure total MV-associated ATP; the second was treated with the apyrase enzyme (2 μg/ml, 30 min, 30 °C) to deplete available ATP outside the MVs and washed with PBS, and then outer-membrane vesicles were lysed with 0.1% Triton X-100 to measure the ATP inside the MVs; in the third, MVs were first lysed with 0.1% Triton X-100 and then treated with apyrase (2 μg/ml, 30 min, 30 °C) to eliminate total ATP. ATP concentration of all three aliquots was measured by the BacTiter-Glo Microbial Cell Viability Assay. *n* = 3. ^*^P < 0.05 vs the other treatments

To confirm the presence of eATP inside MVs, vesicles were collected at OD = 2.2 and split into three equal aliquots. The first one was lysed to make total eATP available for measuring. The second one was treated with the enzyme apyrase to eradicate any free eATP outside the MVs and to measure only the eATP contained in the MVs. Finally, the third aliquot was first lysed and then treated with apyrase to eliminate total eATP. As can be observed in Fig. 4, most ATP was contained inside the MVs (80%) and protected from apyrase treatment.

### Effect of MV Lysis and Apyrase Treatment on *S. vesiculosa* M7^T^ Biofilm Formation

**Fig. 5:**
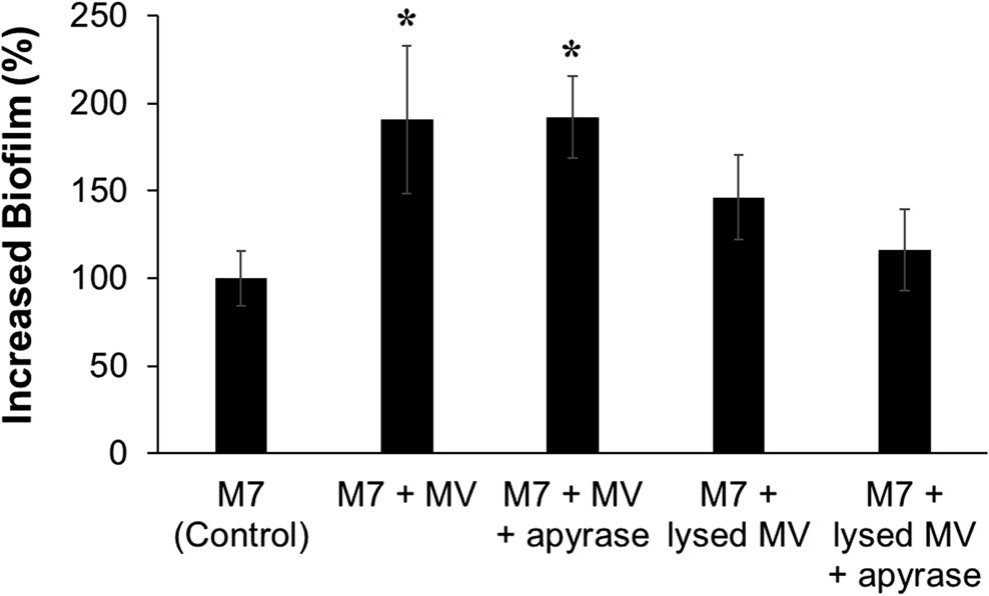
Increase of *S. vesiculosa* M7^T^ biofilm formation due to MV lysis and apyrase treatments. MVs were lysed by 3 freeze-thaw cycles, and apyrase was added at a concentration of 2 μg/ml. Biofilm formation was determined in TSB in 96-well microtiter plates at 15 °C without shaking using the crystal violet method, and the percentage of increase was determined in relation to the M7 strain used as a control. *n* = 2, *P < 0.05 vs M7 control

We explored the effect of breaking MVs and treating them with apyrase. For that purpose, MVs (0.057 mg/ml) were submitted to three freeze-thaw cycles before being added to *S. vesiculosa* M7^T^ cultures. Intact and broken MVs were treated with apyrase, and the effect on biofilm formation was determined (Fig. 5). As observed before, when intact MVs were added to *S. vesiculosa* M7^T^ cultures, the biofilm level increased by 91% in relation to the non-supplemented strain, whereas if MVs were previously lysed to liberate internalized ATP, the biofilm only increased by 45%, constituting a 23.8% reduction in biofilm level with respect to intact MVs. When apyrase was added to the lysed MVs, the biofilm level was reduced by a further 21.6%, whereas the addition of apyrase to intact MVs did not affect the biofilm formation.

## Discussion

Prompted by our previous observation of large amounts of MVs in the extracellular matter of cold-adapted Antarctic bacteria [11], the aim of the present study was to assess the correlation between bacterial MV secretion and biofilm formation, and also evaluate the additional influence of eATP release. Studies have reported the release of eATP during bacterial growth [32, 33] and its presence inside MVs [19].

We found that all the analyzed strains were able to form biofilms in the assayed conditions. One of the most influential factors in biofilm formation is probably the production of exopolymeric substances (EPS) [42]. EPS participate in the different stages of biofilm formation, helping cells to adhere to surfaces, contributing to cell aggregation and biofilm maturation, protecting cells from environmental changes or toxic substances, and allowing nutrient concentration [42]. Colonies from many Antarctic strains have a mucoid appearance, and TEM observations have shown they are able to secrete variable amounts of EPS [11]. This behavior was confirmed in the strains used in the current study, most of which produced EPS observed as a netlike mesh of fibers in the extracellular matter, similar to the observations of other authors [9, 12]. Interestingly, *Pseudomonas* ID1, which showed the greatest ability to form biofilms, produced abundant EPS [34]. Besides cells and EPS, biofilms often contain other components, such as DNA or dead cell debris [8, 43] as well as MVs [13, 14]. Using TEM, we visualized MVs interspersed in the extracellular mucous of all the analyzed strains. However, we did not find a correlation between the ability of the strains to produce MVs and the amount of biofilm obtained.

Although all Gram-negative bacteria are known to produce MVs during growth, the level of production varies, not only within the genus but also within the species [1]. We observed the highest MV release in strains from the genus *Shewanella* and the lowest in the genus *Psychrobacter*, although this behavior was not correlated with an ability to form biofilms. Among the analyzed strains, only *Shewanella vesiculosa* M7^T^ produced large amounts of MVs and had a biofilm-forming capacity that matched the control strain *P. aeruginosa* PAO1. In contrast, another strain from the same genus, *S. livingstonensis* NF22^T^, accumulated the highest concentration of MVs but showed limited biofilm production. Two species of the genus *Psychrobacter*, *P. immobilis* and *P. lutti,* were strong producers of biofilm but secreted very low levels of MVs, and two others, *P. fozzi* and *P. glacincola*, had a low accumulation of MVs and only produced weak biofilms. This discrepancy was also observed in the genera *Pseudomonas* and *Pseudoalteromonas*.

The relationship between biofilms and MVs has been addressed in numerous studies, mainly in pathogenic strains, in an attempt to find ways to block biofilm formation and treat infections [13]. Although several factors have been identified in MVs of particular strains that clearly influence biofilm formation, the complexity of biofilm dynamics and MV composition makes it almost impossible to find a single cause-effect relationship. Changes in the LPS composition, the presence of quorum sensing signals, certain outer membrane proteins, or simply an increased presence of nutrients contributed by MVs are all factors involved in the generation of biofilms [44]. In this work, we evaluated the influence of eATP on biofilm formation. In some cases, eATP induces biofilm dispersion and renders newly formed biofilm more adhesive [25]. It has also been observed that tissues damaged by bacterial infections liberate eATP, which can act as a “danger signal”, eliciting host tissue inflammation and in turn triggering biofilm formation by some pathogenic bacteria to avoid the effects of the immune response [20]. In environmental strains, biofilm formation also increases under conditions of stress, such as temperature changes, lack of nutrients, the presence of toxic compounds, and viral attacks [45, 46]. At the same time, these stress factors can cause an increase in the secretion of MVs, which may be carrying eATP to facilitate biofilm formation.

In most of the Gram-negative Antarctic strains analyzed, we found no correlation between the presence of this extracellular purine and an enhanced biofilm formation. Only *S. vesiculosa* M7^T^ secreted an important amount of eATP and at the same time formed apparent biofilms and produced large amounts of MVs. Consequently, this strain was studied for a potential correlation between these three factors.

*S. vesiculosa* M7^T^ accumulated eATP in the supernatant in a growth-dependent manner, and levels ran parallel to those of icATP, with a peak at the transition between the logarithmic and the stationary growth phases, showing thereafter a marked decline. Our results agree with those reported by Mempin and coworkers [33], although these authors do not describe if the eATP was inside MVs or free in the supernatant. In the case of *S. vesiculosa* M7^T^, we observed the eATP inside MVs, which raises the questions of why and how. It is known that low micromolar concentrations of eATP can influence many biological processes in eukaryotes, and it is becoming increasingly clear that something similar may happen in prokaryotes [30]. For example, eATP regulates the expression of a virulence gene in *Salmonella* [47], and can act as a signaling molecule with significant implications for the development of *P. aeruginosa* biofilms and infections [24]. More recently, it has been demonstrated that eATP is produced in large quantities by the intestinal microbiota, affecting microbiota composition [48].

One explanation for the vesicular packaging of eATP is that it offers protection from extracellular degradation. Abiotic factors such as temperature, pH, pressure, or certain ions can affect eATP stability [49, 50]. In the context of a biofilm, lower levels of eATP could also be due to the presence of other cells that may use it as a source of phosphorous to increase survival or exert other physiological roles. Mempin and coworkers [33] showed that cultures of different bacteria supplemented with ATP had a higher survival rate. Additionally, the presence of active phosphatases in the external environment could potentially cause eATP degradation. Therefore, if eATP does perform important functions, not yet clarified, there are obvious advantages in releasing it in a protected manner, such as inside MVs.

Among the different types of described MVs [3, 51], outer-inner membrane vesicles (O-IMV) could plausibly play a role in the release of a cytoplasmic component such as ATP, as they are formed by protrusion of the inner and outer cell membranes and entrap other cytoplasmic content such as DNA [19, 37]. Another mechanism for the packaging of ATP in MVs of *S. vesiculosa* M7^T^ could be explosive prophage-mediated cell lysis, as proposed by Turnbull et al. [52]. According to this mechanism, the cytoplasmic content can be encapsulated by the recircularization of the cell membranes after lysis. However, more studies are needed to shed light on the origin of eATP in *S. vesiculosa* M7^T^ MVs.

The addition of MVs to cultures of *S. vesiculosa* M7^T^ augmented the rate and amount of biofilm formation. The effects were apparent from the first day, when increasing numbers of small cell clusters or micro-colonies were observed. After day 5 of incubation, there were significant differences in the pattern of adhered cells, with a higher number of large cell clusters, which had the appearance of mature biofilms. It seemed that MVs acted as a nucleation factor in initiating biofilm formation and contributed somehow to increasing the strength of the final biofilm, as described by Schooling and Beveridge [8]. In *S. vesiculosa* M7^T^ cultures, addition of exogenous ATP at the same concentrations as found inside MVs also increased biofilm formation.

It is worth noting that when MVs were broken, their enhancing effect on biofilm formation was no longer significant, indicating that it depended on MV integrity. When ATP was degraded by apyrase treatment of broken MVs, a further reduction in biofilm formation was observed, whereas apyrase treatment of intact MVs had no effect, which points to a role of MV-packaged ATP in biofilm production. MVs from *S. vesiculosa* M7^T^ are complex structures, and components other than ATP may influence biofilm formation, including proteins such as porins, lipidic molecules like LPS, or cytoplasmic components such as DNA [37].

To sum up, MV secretion, biofilm formation, and eATP liberation contribute to bacterial survival, especially in harsh environments such as Antarctica. However, after analyzing a set of Antarctic Gram-negative cold-adapted strains, we found no correlation between these three factors, except in the *S. vesiculosa* M7^T^ strain, in which MVs were found to increase biofilm formation, and eATP packaged inside MVs contributed to this increase. Although the most likely scenario is that the influence of *S. vesiculosa* M7^T^ MVs on biofilm formation is the sum of several factors, further studies should explore the role of eATP once released to the extracellular environment and the mechanisms involved.
